# Role of the Mannose Receptor (CD206) in Innate Immunity to Ricin Toxin

**DOI:** 10.3390/toxins3091131

**Published:** 2011-09-09

**Authors:** Emily Gage, Maria O. Hernandez, Joanne M. O’Hara, Elizabeth A. McCarthy, Nicholas J. Mantis

**Affiliations:** 1 Division of Infectious Disease, Wadsworth Center, New York State Department of Health, Albany, NY 12208, USA; Email: gage.emily@gmail.com (E.G.); moh01@wadsworth.org (M.O.H.); joanne.m.ohara@gmail.com (J.M.O.); emccart2@gmail.com (E.A.M.); 2 Department of Biomedical Sciences, University at Albany School of Public Health, Albany, NY 12201, USA

**Keywords:** toxin, pathogenesis, *C*-type lectins, biodefense

## Abstract

The entry of ricin toxin into macrophages and certain other cell types in the spleen and liver results in toxin-induced inflammation, tissue damage and organ failure. It has been proposed that uptake of ricin into macrophages is facilitated by the mannose receptor (MR; CD206), a *C*-type lectin known to recognize the oligosaccharide side chains on ricin’s A (RTA) and B (RTB) subunits. In this study, we confirmed that the MR does indeed promote ricin binding, uptake and killing of monocytes *in vitro*. To assess the role of MR in the pathogenesis of ricin *in vivo*, MR knockout (MR^−/−^) mice were challenged with the equivalent of 2.5× or 5× LD_50_ of ricin by intraperitoneal injection. We found that MR^−/−^ mice were significantly more susceptible to toxin-induced death than their age-matched, wild-type control counterparts. These data are consistent with a role for the MR in scavenging and degradation of ricin, not facilitating its uptake and toxicity *in vivo*.

## 1. Introduction

Ricin toxin, a natural product of the castor bean plant (*Ricinus communis*), is one of the most potent toxins known. Ricin is capable of killing virtually all mammalian cell types, and can be lethal to humans following injection, inhalation or ingestion. The study of ricin has led to the discovery of fundamental processes in areas of protein science, cell biology and immunology, and has had important applications for immunotherapeutics [1,2,3]. At the same time, ricin has sordid history as a biological weapon. During World War II, ricin was weaponized under the code name “Compound W” by the US military, although the toxin was never used in combat [4,5]. The KGB employed a ricin-laced metal pellet in the London assassination of the Bulgarian dissident Georgi Markov in 1978, and the toxin remains one of the more common “white powders” encountered by law enforcement [6,7]. For these reasons, the Centers for Disease Control have classified ricin as a Category B Select Agent, and the National Institutes of Health and (NIAID) considers the development of countermeasures against ricin a research priority [8].

Ricin is a member of the so-called A-B family of toxins. The toxin consists of a 267 amino acid A subunit (RTA) and 262 amino acid B subunit (RTB) that are joined via a single disulfide bond. RTA is a ribosome inactivating protein (RIP) whose exclusive substrate is a universally conserved adenosine moiety within the so-called sarin/ricin loop (SRL) of eukaryotic ribosomal RNA [9]. RTA is so potent that a single molecule is sufficient to kill a cell [10]. Ricin’s B subunit (RTB) is a lectin specific for β-1,3-linked galactose and *N*-acetylgalactosamine (Gal/GalNac) residues on both glycolipids and glycoproteins [11]. Both RTA and RTB are post-translationally modified via *N*-linked mannose side chains. RTA has at least one modification at position Asn10 (GlcNAc_2_Man_4_), while RTB has two modifications at positions Asn95 (GlcNAc_2_Man_6_) and Asn135 (GlcNAc_2_Man_7_) [12,13].

The pathogenesis of ricin toxin has been studied in small animal models, especially rodents [14]. Following intravenous administration, ricin is cleared from the circulation and accumulates primarily within the liver and spleen, although residual amounts of the toxin are also found in the lungs, kidneys and intestines [15,16,17,18,19,20]. Within the spleen and liver, macrophages (MΦ) cells are the first (and most severely) affected by the toxin. As early as four hours post challenge, Kupffer cells exhibit ultrastructural changes consistent with the initiation of apoptosis [15]. In parallel, toxin-mediated tissue damage is accompanied by a severe inflammatory response, which has been postulated to originate from MΦs [16,21]. Consistent with this model is the fact that MΦs exposed to ricin *in vitro* results in the activation of stress-activated protein kinases and secretion of an array of pro-inflammatory cytokines and chemokines [22,23,24,25].

Macrophages are proposed to internalize ricin by two distinct pathways [26,27]. The first pathway involves RTB-mediated attachment to galactosyl residues on surface localized glycoproteins and glycolipids. Once bound to the cell surface via RTB, ricin is endocytosed and trafficked retrograde to the endoplasmic reticulum (ER), where RTA dissociates from RTB and is retro-translocated across the ER membrane into the cytosol [3,28]. This pathway is relatively inefficient in that only a fraction (<5%) of the total amount of RTA internalized by this route gains access to the TGN; the vast majority of ricin is recycled to the cell surface or degraded [29]. On the other hand, the pathway is largely insaturable, as it is estimated that there are more than 1 × 10^6–7^ RTB bindings sites on the surface of a typical cell [30]. The second, and biochemically distinct, uptake pathway involves the mannose receptor (MR) [26,27,31]. The MR, also known as CD206, is a 180 kDa transmembrane *C*-type lectin that binds oligosaccharides terminating in mannose, fucose or *N*-acetylglucosamine [32,33]. Simmons and colleagues first demonstrated that ricin uptake into rat peritoneal macrophages was reduced by either addition of exogenous lactose or mannan, and that maximal inhibition occurred when both lactose and mannan were present [27]. At the time, the only described mannose binding protein on peritoneal macrophages was the MR; a number of other mannose-specific lectins have since been identified [34].

Despite the potential importance of the MR in ricin pathogenesis, the contribution of the MR to ricin intoxication *in vivo* has never been directly examined. Studies aimed at addressing this issue have relied on indirect means of blocking MR activity, such as co-injection of high concentrations of mannan with ricin in an attempt to saturate endogenous MR activity [31]. While these studies strongly support a role for the MR in ricin uptake *in vivo*, they are confounded by the possibility that insufficient mannan was present to fully saturate the MR, or that other *C*-type lectins (e.g., DC-SIGN) in addition to the MR may be involved in ricin recognition and uptake. Defining the exact role of the MR in ricin pathogenesis would be most easily addressed through the use knockout mice specifically deficient in the MR. In fact, the laboratory of Dr. Michel Nussenzweig recently produced and characterized MR knock out (MR^−/−^) mice [35]. The MR^−/−^ mice were demonstrably impaired in their ability to clear serum glycoproteins, a phenotype that is consistent a role for the MR in promoting the recognition, degradation and excretion of endogenously- and exogenously-derived mannosylated-ligands.

Thus, the overarching goal of this study was to take advantage of the availability of MR^−/−^ mice to directly determine the role of the MR in mediating the pathogenesis of ricin *in vivo*. If the MR functions in facilitating ricin uptake into MΦs or other cell types, then we would expect that MR^−/−^ mice will be more resistant to ricin challenge than their wild type counterparts. On the other hand, if the MR promotes the clearance and/or degradation of ricin from circulation, as it does for other serum glycoproteins, then MR^−/−^ mice would be expected to be more sensitive to ricin challenge. In this study, we first confirm that the MR does indeed promote the binding and uptake of ricin into MΦs *in vitro*. We then perform ricin challenge studies with wild type and MR^−/−^ mice and demonstrate that the MR^−/−^ mice are in fact more sensitive to ricin than wild type control animals. These and other results strongly suggest that the MR is ultimately involved in innate immunity to ricin, probably through the capacity of the receptor to accelerate ricin clearance and degradation. 

## 2. Materials and Methods

### 2.1. Chemicals, Reagents and Antibodies

Ricin and ricin conjugated to fluorescein isothiocyanate (FITC) were purchased from Vector Laboratories (Burlingame, CA). Ricin was dialyzed against PBS at 4 °C in 10,000 MW cutoff Slide-A-Lyzer dialysis cassettes (Pierce, Rockford, IL), prior to use in cytotoxicity and animal studies. Fluorophore- and biotin-conjugated monoclonal antibodies (mAbs) directed against the human (clone 15–2) and mouse (clone MR5D3) mannose receptors were purchased from BioLegend (San Diego, CA). Yeast mannan was obtained from Sigma-Aldrich (St. Louis, MO). Cell culture media were prepared by the Wadsworth Center Media Services facility. 

### 2.2. Cytotoxicity and Apoptosis Assays

THP-1 cells are a monocytic leukemic cell line [36] that we obtained from the American Type Culture Collection (ATCC, Manassas, VA). The cells were passaged in complete RPMI containing 10% fetal bovine serum and were maintained in a humidified incubator (37 °C, 5% CO_2_). For cytotoxicity assays, the cells were adjusted to 5 × 10^4^ cells per mL, seeded (100 μL/well) onto white 96 well plates (Corning Life Sciences, Lowell, MA). Twenty-four hours later, the cells were treated with ricin (50 ng/mL) for 2 h at 37 °C. The cells were then washed to remove unbound toxin and incubated for an additional 40 h. Cell viability was assessed using CellTiter-GLO reagent (Promega, Madison, WI) according to the manufacturer’s instructions, except the reagent was diluted 1:5 in PBS before use. Luminescence was measured with a SpectraMax L luminometer interfaced with SoftMax Pro software (version 5.2; Molecular Devices, Sunnyvale, CA). All treatments were performed in triplicate and 100% viability was defined as the average value obtained from wells treated with medium only. 

Annexin-V staining was used as a surrogate marker of cells undergoing apoptosis [37]. THP-1 cells (1 × 10^6^ cells per mL) were seeded into 12 well cell culture plates (Corning). The cells were then incubated at 37 °C with ricin (1 μg/mL), ricin-mAb mixtures or ricin-polysaccharide mixtures for 4.5 hours. After incubation, the cells were collected by centrifugation and then re-suspended in 1X binding buffer (100 μL) containing 5 μL of Annexin V-FITC and 5 μL PI, as recommended by the manufacturer (BD Biosciences). Samples were assayed for apoptosis and necrosis using a FACS Calibur (BD Biosciences). Results were reported as % cells positive for Annexin V-FITC or PI. A minimum of 20,000 cells were analyzed per sample.

### 2.3. Toxin Binding Assays

To examine ricin binding to cell surfaces, THP-1 monocyte cells were washed and then resuspended in 4 °C HBSS at a concentration of 1 × 10^6^ mL. The cells were then chilled on ice for 20 min and then incubated with FITC-labeled ricin (1 μg/mL) in the presence of specific mAbs or sugars for 20 min. The cells were then washed with HBSS to remove unbound ricin-FITC, fixed in 1% paraformaldehyde for 10 min, and resuspended in PBS with 2% goat serum (Invitrogen) containing 1 mM NaN_3_. The cells were subjected to flow cytometry using a FACSCalibur (BD Biosciences). A minimum of 20,000 cells were analyzed per sample. 

### 2.4. Animals and Ricin Challenge Studies

MR^−/−^ breeder pairs were kindly provided by the laboratory of Dr. Michel Nussenzweig (Rockefeller University, New York, NY) [35]. Animals were housed under conventional, specific pathogen-free conditions and were treated in compliance with the Wadsworth Center’s Institutional Animal Care and Use Committee (IACUC) guidelines. For challenge studies, ricin was diluted into DPBS and administered by intraperitoneal injection to groups of MR^−/−^ mice (8–12 weeks of age) and gender- and age-matched C57/B6 control mice. Survival was monitored over a 3-day period. Hypoglycemia was used as a surrogate marker of intoxication. Blood glucose levels were measured using an Aviva ACCU-CHECK handheld blood glucose meter (Roche, Indianapolis, IN). Mice were euthanized when their blood glucose levels fell below 25 mg/dL. For statistical purposes, readings at or below the meter’s limit of detection of ~12 mg/dL were set to that value. 

To estimate the half-life of ricin in serum, groups of age- and gender-matched wild type and MR^−/−^ mice injected intravenously with biotinylated ricin (100 μg/mouse). Blood was collected from the animals 30 min and 60 min later by intra-orbital bleed. Ricin levels in serum were determined by ELISA in which NUNC Maxisorb F96 microtiter plates (Thermo Fisher Scientific, Waltham, MA) were coated overnight with the RTB-specific mAb 24B11 (1 μg/mL) [38] to capture ricin, treated with sera from biotin-ricin challenged mice, and then horseradish peroxidase (HRP)-labeled avidin (Sigma Aldrich Co., St. Louis, MO). The plates were developed using the colorimetric detection substrate 3,3′,5,5′-tetramethylbenzidine (TMB; Kirkegaard & Perry Labs, Gaithersburg, MD) and were analyzed with a SpectroMax 250 spectrophotometer. 

To further investigate leukocyte apoptosis in the spleens of ricin challenged mice, groups of MR^−/− ^mice and gender- and age-matched C57/B6 control mice were challenged with ricin (1 μg) by IP injection. Eighteen hours later, the animals were euthanized and spleenocytes were physically dissociated in DMEM +10% FBS. After centrifugation (400 × g) for 5 min, the cells were then incubated in 0.17 M ammonium chloride (pH 7.4) for 10 min to lyse red blood cells. After incubation, the cells were collected by centrifugation and then suspended in 1× binding buffer (100 μL) containing 5 μL of Annexin V-FITC and 5 μL PI, as recommended by the manufacturer (BD Biosciences). Samples were assayed for apoptosis and necrosis using a FACS Calibur (BD Biosciences). 

Levels of the pro-inflammatory cytokine and chemokines γ-interferon, interleukin (IL)-1, IL-6, IL-12p70, monocyte chemotactic protein 1 (MCP-1), and tumor necrosis factor (TNF)-α levels in splenic homogenates were determined using a BD CBA Mouse Inflammation kit (BD Biosciences). The cytokine/chemokine concentrations were calculated from standard curves generated from purified cytokines/chemokines provided by the manufacturer. 

### 2.5. Statistical Analysis and Software

Statistical analysis was carried out with GraphPad Prism 5 (GraphPad Software, San Diego, CA). For survival studies, statistical significance between groups was determined using the Log-Rank (Mantel-Cox) test. Differences in blood glucose levels and cytokine levels were determined by two-way analysis of variance (ANOVA).

## 3. Results

### 3.1. MR Contributes to Ricin-Induced Killing of Monocytes *in Vitro*

**Figure 1:**
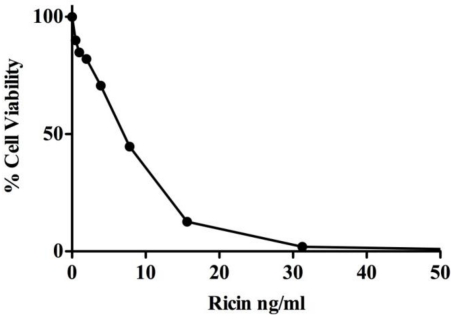
Sensitivity of THP-1 cells to ricin toxin. THP-1 cells (5 × 10^4^ cells per mL) were treated with indicated concentrations of ricin for 2 h at 37 °C. The cells were then washed to remove unbound toxin and incubated for an additional 40 h. Cell viability was assessed using as described in Materials and Methods.

THP-1 cells are a human acute monocytic leukemia cell line used widely as a model for monocyte-derived macrophages [36]. These cells are known to express a number of *C*-type lectins, including the MR [39,40,41], even in their undifferentiated state. Furthermore, these cells are highly sensitive to the effects of ricin (Figure 1). 

**Figure 2:**
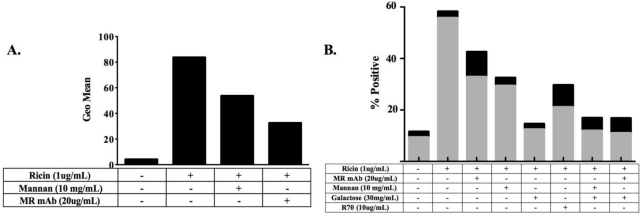
Contribution of the Mannose Receptor (MR) in ricin binding and uptake by THP-1 cells. (**A**) Role of MR in ricin attachment. THP-1 cells (1 × 10^6^ cells/mL) were chilled on ice for 20 minutes to arrest endocytosis. Cells were then treated with FITC-labeled ricin (1 μg/mL) in the presence of mannan (10 mg/mL) or the blocking anti-MR mAb 15–1 for 20 min before being subjected to flow cytometric analysis. Geo Mean refers to the geometric mean fluorescence emission at 519 nm when cells were exposed to an excitation signal 488 nm; and (**B**) Role of the MR in ricin cytotoxicity. THP-1 cells (1 × 10^6^ cells per mL) were incubated with ricin (1 μg/mL), ricin-mAb mixtures, or ricin-sugar (mannan or galactose) mixtures 4.5 h. After which the cells were assayed for Annexin-V expression (gray bars) as an indicator of apoptosis and PI staining (black bars) as an indicator of necrosis. The Y-axis refers to % cells that were positive for Annexin V-FITC or PI. A minimum of 20,000 cells were analyzed per sample.

We therefore used these cells as a model system to verify previous reports demonstrating a role for the MR in ricin uptake and toxicity [27]. To assess the role of the MR in promoting ricin attachment to cells, THP-1 cells were exposed to FITC-ricin (1 μg/mL) at 4 °C for 20 min, washed, fixed, and then subjected to flow cytometry. These studies were conducted at 4 °C to permit binding to cells, but not endocytosis [42]. FITC-ricin bound cells with a MFI of ~80 (Figure 2A). Toxin binding to cell surfaces was reduced by ~25% upon the addition of mannan (10 mg/mL) and more than 50% by treatment of the cells with a MR-specific blocking mAb. Activation of the THP-1 cells with IL-4 resulted in increased surface expression of the MR and a concomitant increase in ricin binding that was also partially inhibited by mannan and the MR-specific blocking mAb (data not shown). These results confirm a role for the MR in toxin attachment to cell surfaces.

To define the role of the MR in ricin toxicity, cells were exposed to ricin (1 μg/mL) for 5 h and then assessed for apoptosis by staining the cells with annexin-V. Following ricin treatment, approximately 60% of the cells stained positive for annexin V, indicating that apoptosis had been initiated in a majority of the cells population (Figure 2B). The addition of anti-MR mAb (20 μg/mL) or excess mannan (10 mg/mL) partially protected the cells from the effects of ricin, consistent with a role for the MR in ricin uptake. The degree of protection conferred by the anti-MR mAb and mannan was only slightly less than that conferred by the well-characterized neutralizing mAb R70 [38,43]. Interestingly, the addition of galactose virtually eliminated ricin-induced cell killing, underscoring the contribution of RTB-mediated uptake via terminal galactose residues in ricin entry into host cells. The combination of galactose and mannan was not more effective than galactose alone in preventing toxin-induced killing, which has implications for the role of MR in ricin uptake (see below). 

### 3.2. Sensitivity of MR^−/−^ Mice to Ricin Intoxiction

Having confirmed a role for the MR in the binding to and uptake by a monocyte cell line *in vitro*, we next wished to examine the contribution of the MR to *in vivo*. We obtained MR^−/−^ breeder pairs from the laboratory of Dr. Michel Nussenzweig at the Rockefeller University [35]. Groups of age-, weight and sex-matched C57BL/6 and MR^−/−^ mice were challenged with 1.0 μg or 0.5 μg of ricin, which in wild type mice is the equivalent of 5× LD_50_ or 2.5× LD_50_, respectively. Mice were then monitored for morbidity and mortality for three days. Hypoglycemia was used as a quantitative surrogate marker of ricin intoxication [44].

**Figure 3:**
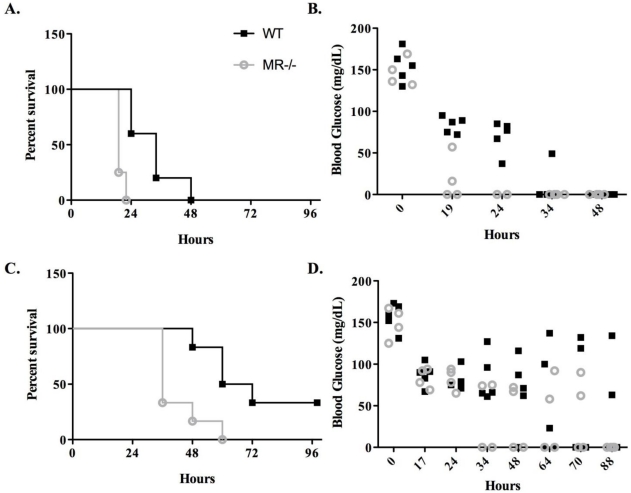
Survival of MR^−/−^ mice following ricin challenge. Groups of MR^−/−^ or age- and sex-matched wild type controls were challenged with 1.0 μg (panels A, B) or 0.5 μg (panels C, D) ricin. Groups of animals were monitored for survival (A, C) and hypoglycemia (B, D). Panels A, C: At both challenge doses, the MR^−/−^ mice succumbed to ricin intoxication at a rate significantly greater than their wild type counterparts (*p* < 0.01 by the Log-Rank test). Panels B and D: each symbol represents an individual mouse. For mice challenged with 1.0 μg ricin (panel A), blood glucose levels in the MR^−/−^ mice were significantly lower that their wild type counterparts at 19 h (*p* < 0.001) and 24 h (*p* < 0.001) post challenge, as determined by two-way ANOVA. For mice challenged with 0.5 μg ricin (panel B), blood glucose levels in the MR^−/−^ mice tended to be lower than wild type controls, although this difference was not statistically significant.

Wild-type mice challenged with 1 μg of ricin (equivalent to 5× LD_50_) succumbed to intoxication within 48 h, with a median survival of 34 h (Figure 3A). In contrast, all the MR^−/−^ mice challenged with the same amount of toxin expired within 24 h, with a median survival of 19 h (Figure 3A). The enhanced susceptibility of the MR^−/−^ mice to ricin intoxication was also apparent in the groups of mice challenged with 0.5 μg: wild-type mice had a median survival of 66 h, whereas the MR^−/−^ mice had a median survival of only 36 h (Figure 3B). The kinetics of hypoglycemia mirrored the time to death curves for both groups of animals. (Figure 3C,D) In both the high and low ricin challenge groups, the MR^−/−^ mice demonstrated a more rapid reduction in blood glucose levels as compared to their wild type counterparts. These data reveal that the MR^−/−^ mice are approximately two-times more sensitive to ricin than wild type mice. 

**Figure 4:**
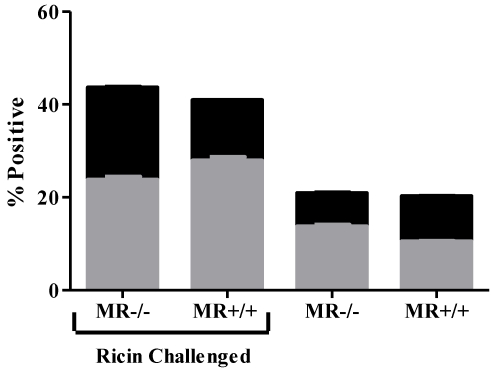
Toxin-induced apoptosis in the spleens of MR^−/−^ and wild type mice. Splenocytes from wild type and MR^−/−^ control and ricin-challenged mice (18 h post challenge) were subjected to Annexin V-FITC (gray bars) and PI staining (black bars) as indicators of apoptosis and necrosis, respectively. There was no significant difference in apoptosis numbers between the wild type and mannose receptor-deficient animals.

To examine whether the serum half-life of ricin was affected by the absence of the MR, groups of wild type or knock-out mice were administered ricin intravenously. Toxin levels in serum were determined by ELISA 30 and 60 min later. By this method, there was no detectable difference in the clearance of ricin from circulation between the wild-type and MR^−/−^ mice (data not shown). Furthermore, the levels of ricin-induced apoptosis in the spleens wild-type and MR^−/−^ mice were identical (Figure 4), indicating that equal amounts of toxin likely gained access to this (and other) visceral organs. 

**Figure 5:**
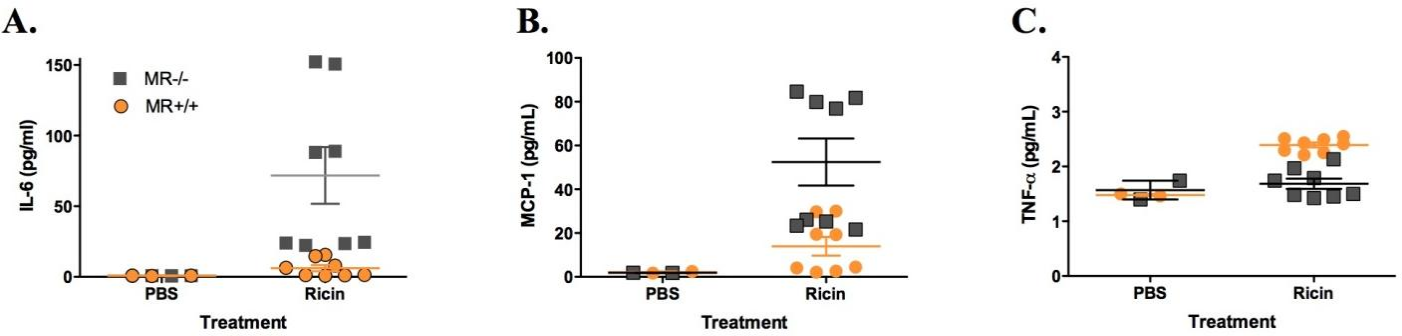
Cytokine and chemokine levels in splenic homogenates of MR^−/−^ and wild type mice following ricin challenge. Splenic homogenates from groups of wild type and MR^−/−^ control and ricin-challenged mice (18 h post challenge) were subjected mouse inflammatory cytokine/chemokine cytometric bead array. Shown are values from IL-6 (A), MCP-1 (B), and TNF-α (C). The differences in IL-6 levels in ricin-challenged MR^−/−^ mice were significantly greater than those in ricin-challenged control animals (*p* < 0.05, as determined by ANOVA).

Interestingly, however, IL-6 levels in the spleens of ricin-challenged MR^−/−^ mice were 2–6 fold higher than was observed in the ricin-challenged wild type controls (Figure 5). Other cytokines, notably MCP-1, TNF-α, IL-1 and IFN-γ were altered but not significantly different between the groups of animals (Figure 5C, data not shown). These data demonstrate that the absence of the MR does not affect toxin-induced cell death in the spleen, but does influence pro-inflammatory cytokine/chemokne responses in the serum. 

Finally, we also examined pro-inflammatory cytokine/chemokine responses in the intestinal mucosa in MR^−/−^ mice following an intragastric ricin challenge. We previously reported that MCP-1 (but not TNF-α, IL-1, IL-6 or IFN-γ) is a hallmark of ricin-induced damage to the intestinal mucosa [45]. Groups of wild-type and MR^−/−^ mice were challenged intragastrically with 5 mg/kg of ricin. Approximately 24 h later, the animals were euthanized and intestinal homogenates were assayed by CBA analysis for cytokine/chemokine levels. MCP-1 levels were elevated in ricin-challenged mice, as compared to sham challenged controls (data not shown). There was no difference, however, in MCP-1 levels at baseline or following toxin challenge between wildtype and MR^−/−^ mice.

## 4. Conclusions and Discussion

In this study, we took advantage of the availability of MR^−/−^ mice to examine the role of MR-mediated uptake in the pathogenesis of ricin toxin *in vivo*. We found that MR^−/−^ mice were approximately two-fold more sensitive to ricin-induced killing than their wild-type counterparts. MR^−/−^ mice challenged with the equivalent of 2.5× or 5× LD_50_ of ricin demonstrated a more rapid reduction in blood glucose levels and an accelerated time to death, as compared to control animals. While these data are surprising in light of the fact that the MR actually promotes ricin-induced apoptosis of monocytes/macrophages *in vitro*, they are entirely consistent with the MR’s established role as a scavenging receptor and an important component of the innate immune system [32,33,35]. Thus, we propose that MR-mediated uptake of ricin by macrophages (and other cell types) *in vivo* results in the clearance and degradation of the toxin, and does not lead to an increase in toxin-induced cell death or an increased inflammatory response. In the absence of the MR, ricin internalization likely occurs entirely through the RTB-mediated uptake pathway.

As a member of the *C*-type lectin family of pattern recognition receptors (PRRs), the MR has been shown to bind (and in some cases mediate phagocytosis of) a variety of bacterial and fungal pathogens, including *Mycobacterium tuberculosis* [46], *Streptococcus pneumoniae* [47], *Francisella tularensis* [48], *Pneumocystis carinii* [49], *Candida albicans* [50], and *Cryptococcus neoformans* [51]. It has been postulated that microbial pathogens exploit the MR as a means to gain entry into host cells. With the exception of *F. tularensis* [48], however, this postulate has not been substantiated. For example, mice lacking the MR are more sensitive (not more resistant) to *Cryptococcus neoformans* infection than wild type animals, as reflected by faster time to death and higher fungal burdens following pulmonary challenge [51]. Thus, it is generally accepted that the primary function of the MR is in antigen capture and degradation. Indeed, the MR may be integral in linking the innate and adaptive immune responses, as evidenced by the fact that MR-mediated uptake of certain exogenous glycoproteins by dendritic cells results in cross-presentation to CD8^+^ T cells [52]. While our data clearly support a role for the MR in the clearance of ricin from circulation, it remains to be determined whether the MR also contributes to mounting an immune response to the toxin. 

It is interesting to note that, in our hands, ricin attachment and uptake into monocytes/macrophages occurs primarily via the RTB-mediated pathway. For example, treatment of THP-1 cells with mannan or a MR blocking antibody reduced ricin binding to cell surfaces by 20–50%. In contrast, binding was reduced by more than 80% with the addition of lactose. The addition of mannan did not reduce toxin binding beyond that observed with lactose alone. Based on these observations, we postulate that ricin uptake via the MR may occur in a two-step fashion. Specifically, we propose that ricin may first adhere to cell surfaces via RTB’s affinity for Gal/GalNac. Once bound to the cell surface, ricin may associate with the MR via mannose side chains on RTA and/or RTB. This model would explain our observation that monoclonal antibodies that block RTB’s galactose recognition activity (but do not affect accessibility of the mannose side chains) completely neutralize ricin *in vitro* [38], block attachment of ricin to primary macrophages *ex vivo*, and protect mice against a lethal ricin challenge (A. Yermakova and N. Mantis, in press). 

In conclusion, we have demonstrated that MR^−/−^ mice are significantly more susceptible to ricin-induced intoxication than age-matched, wild-type counterparts. These data are consistent with the MR contributing to ricin recognition *in vivo*, but indicate that the ricin’s fate following uptake via the MR is one of degradation and not intoxication. Thus, the fate of ricin following MR uptake may differ *in vitro* (or *ex vivo*) versus *in vivo*. Why this is the case is not immediately apparent, but may be due in part to influences by local environments *in vivo* that are not recapitulated *in vitro*. It should be stressed, however, that the actual fate of ricin in the MR^−/−^ mice following intraperitoneal injection was not determined in this study. Thus, while we propose that the susceptibility of the MR^−/−^ mice to ricin is due to the failure of macrophages to efficiently clear the toxin from circulation, there are other possible explanations. For example, we cannot exclude the possibility that soluble MR or other serum proteins contribute to the scavenging ricin in circulation and/or interstitial fluids (e.g., in the peritoneal cavity) [53]. Nor can we ignore the observation that MR^−/−^ mice have elevated serum levels of at least three proteins associated with inflammation and wound healing [35] that could possibility interfere directly or indirectly with ricin toxicity. Future studies will be aimed at defining the exact role for MR in the clearance of ricin *in vivo* and determining how this activity might be exploited in developing countermeasures against this toxin. 

## References

[1] Audi J., Belson M., Patel M., Schier J., Osterloh J. (2005). Ricin poisoning: A comprehensive review. J. Am. Med. Assoc..

[2] Olsnes S. (2004). The history of ricin, abrin and related toxins. Toxicon.

[3] Sandvig K., van Deurs B. (2005). Delivery into cells: Lessons learned from plant and bacterial toxins. Gene. Ther..

[4] Franz D., Jaax N., Zajtchuk R.B. (1997). Ricin Toxin. Textbook of Military Medicine.

[5] Maman M., Yehezkelli Y., Fong S., Alibek K. (2005). Ricin: A Possible, Non-Infectious Biological Weapon. Bioterrorism and Infectious Agents.

[6] Hulse C. (2004). Tests indicate poison in senate mail room of majority leader. N. Y. Times.

[7] Schier J.G., Patel M.M., Belson M.G., Patel A., Schwartz M., Fitzpatrick N., Drociuk D., Deitchman S., Meyer R., Litovitz T., Watson W.A., Rubin C.H., Kiefer M. (2007). Public health investigation after the discovery of ricin in a South Carolina postal facility. Am. J. Public Health.

[8] (2004). Summary of the NIAID Ricin Expert Panel Workshop.

[9] Endo Y., Mitsui K., Motizuki M., Tsurugi K. (1987). The mechanism of action of ricin and related toxins on eukaryotic ribosomes. J. Biol. Chem..

[10] Eiklid K., Olsnes S., Pihl A. (1980). Entry of lethal doses of abrin, ricin and modeccin into the cytosol of HeLa cells. Exp. Cell Res..

[11] Baenziger J.U., Fiete D. (1979). Structural determinants of Ricinus communis agglutinin and toxin specificity for oligosaccharides. J. Biol. Chem..

[12] Foxwell B.M., Donovan T.A., Thorpe P.E., Wilson G. (1985). The removal of carbohydrates from ricin with endoglycosidases H, F and D and alpha-mannosidase. Biochim. Biophys. Acta.

[13] Kimura Y., Hase S., Kobayashi Y., Kyogoku Y., Ikenaka T., Funatsu G. (1988). Structures of sugar chains of ricin D. J. Biochem. (Tokyo).

[14] Bradberry S.M., Dickers K.J., Rice P., Griffiths G.D., Vale J.A. (2003). Ricin poisoning. Toxicol. Rev..

[15] Bingen A., Creppy E.E., Gut J.P., Dirheimer G., Kirn A. (1987). The Kupffer cell is the first target in ricin-induced hepatitis. J. Submicrosc. Cytol..

[16] Derenzini M., Bonetti E., Marionozzi V., Stirpe F. (1976). Toxic effects of ricin: Studies on the pathogenesis of liver lesions. Virchows Arch. B.

[17] Fodstad O., Olsnes S., Pihl A. (1976). Toxicity, distribution and elimination of the cancerostatic lectins abrin and ricin after parenteral injection into mice. Br. J. Cancer.

[18] Skilleter D.N., Foxwell B.M. (1986). Selective uptake of ricin A-chain by hepatic non-parenchymal cells *in vitro*. Importance of mannose oligosaccharides in the toxin. FEBS Lett..

[19] Thorpe P.E., Detre S.I., Foxwell B.M., Brown A.N., Skilleter D.N., Wilson G., Forrester J.A., Stirpe F. (1985). Modification of the carbohydrate in ricin with metaperiodate-cyanoborohydride mixtures. Effects on toxicity and *in vivo* distribution. Eur. J. Biochem..

[20] Zenilman M.E., Fiani M., Stahl P., Brunt E., Flye M.W. (1988). Use of ricin A-chain to selectively deplete Kupffer cells. J. Surg. Res..

[21] Brown R.F., White D.E. (1997). Ultrastructure of rat lung following inhalation of ricin aerosol. Int. J. Exp. Path..

[22] Gonzalez T.V., Farrant S.A., Mantis N.J. (2006). Ricin induces IL-8 secretion from human monocyte/macrophages by activating the p38 MAP kinase pathway. Mol. Immunol..

[23] Higuchi S., Tamura T., Oda T. (2003). Cross-talk between the pathways leading to the induction of apoptosis and the secretion of tumor necrosis factor-alpha in ricin-treated RAW 264.7 cells. J. Biochem. (Tokyo).

[24] Korcheva V., Wong J., Lindauer M., Jacoby D.B., Iordanov M.S., Magun B. (2007). Role of apoptotic signaling pathways in regulation of inflammatory responses to ricin in primary murine macrophages. Mol. Immunol..

[25] Licastro F., Morini M.C., Bolognesi A., Stirpe F. (1993). Ricin induces the production of tumour necrosis factor-alpha and interleukin-1 beta by human peripheral-blood mononuclear cells. Biochem. J..

[26] Frankel A.E., Fu T., Burbage C., Tagge E., Harris B., Vesely J., Willingham M.C. (1997). Lectin-deficient ricin toxin intoxicates cells bearing the D-mannose receptor. Carbohydr. Res..

[27] Simmons B.M., Stahl P.D., Russell J.H. (1986). Mannose receptor-mediated uptake of ricin toxin and ricin A chain by macrophages. Multiple intracellular pathways for a chain translocation. J. Biol. Chem..

[28] Spooner R.A., Lord J.M. (2011). How ricin and shiga toxin reach the cytosol of target cells: Retrotranslocation from the endoplasmic reticulum. Curr. Top. Microbiol. Immunol..

[29] Van Deurs B., Sandvig K., Petersen O.W., Olsnes S., Simons K., Griffiths G. (1988). Estimation of the amount of internalized ricin that reaches the trans-Golgi network. J. Cell Biol..

[30] Sandvig K., Olsnes S., Pihl A. (1976). Kinetics of binding of the toxic lectins abrin and ricin to surface receptors of human cells. J. Biol. Chem..

[31] Magnusson S., Berg T. (1993). Endocytosis of ricin by rat liver cells *in vivo* and *in vitro* is mainly mediated by mannose receptors on sinusoidal endothelial cells. Biochem. J..

[32] East L., Isacke C.M. (2002). The mannose receptor family. Biochim. Biophys. Acta.

[33] Taylor P.R., Gordon S., Martinez-Pomares L. (2005). The mannose receptor: Linking homeostasis and immunity through sugar recognition. Trends Immunol..

[34] Kerrigan A.M., Brown G.D. (2009). *C*-type lectins and phagocytosis. Immunobiology.

[35] Lee S.J., Evers S., Roeder D., Parlow A.F., Risteli J., Risteli L., Lee Y.C., Feizi T., Langen H., Nussenzweig M.C. (2002). Mannose receptor-mediated regulation of serum glycoprotein homeostasis. Science.

[36] Auwerx J. (1991). The human leukemia cell line, THP-1: A multifacetted model for the study of monocyte-macrophage differentiation. Experientia.

[37] Van Engeland M., Ramaekers F.C., Schutte B., Reutelingsperger C.P. (1996). A novel assay to measure loss of plasma membrane asymmetry during apoptosis of adherent cells in culture. Cytometry.

[38] McGuinness C.R., Mantis N.J. (2006). Characterization of a novel high-affinity monoclonal immunoglobulin G antibody against the ricin B subunit. Infect. Immun..

[39] Baumann J., Park C.G., Mantis N.J. (2010). Recognition of secretory IgA by DC-SIGN: Implications for immune surveillance in the intestine. Immunol. Lett..

[40] Diaz-Silvestre H., Espinosa-Cueto P., Sanchez-Gonzalez A., Esparza-Ceron M.A., Pereira-Suarez A.L., Bernal-Fernandez G., Espitia C., Mancilla R. (2005). The 19-kDa antigen of Mycobacterium tuberculosis is a major adhesin that binds the mannose receptor of THP-1 monocytic cells and promotes phagocytosis of mycobacteria. Microb. Pathog..

[41] Puig-Kroger A., Serrano-Gomez D., Caparros E., Dominguez-Soto A., Relloso M., Colmenares M., Martínez-Muñoz L., Longo N., Sánchez-Sánchez N., Rincon M., Rivas L., Sánchez-Mateos P., Fernández-Ruiz E., Corbí A.L. (2004). Regulated expression of the pathogen receptor dendritic cell-specific intercellular adhesion molecule 3 (ICAM-3)-grabbing nonintegrin in THP-1 human leukemic cells, monocytes, and macrophages. J. Biol. Chem..

[42] Sandvig K., Olsnes S. (1979). Effect of temperature on the uptake, excretion and degradation of abrin and ricin by HeLa cells. Exp. Cell Res..

[43] Neal L.M., O’Hara J., Brey R.N., Mantis N.J. (2010). A monoclonal immunoglobulin G antibody directed against an immunodominant linear epitope on the ricin A chain confers systemic and mucosal immunity to ricin. Infect. Immun..

[44] Pincus S.H., Eng L., Cooke C.L., Maddaloni M. (2002). Identification of hypoglycemia in mice as a surrogate marker of ricin toxicosis. Comp. Med..

[45] Yoder J.M., Aslam R.U., Mantis N.J. (2007). Evidence for widespread epithelial damage and coincident production of monocyte chemotactic protein 1 in a murine model of intestinal ricin intoxication. Infect. Immun..

[46] Kang B.K., Schlesinger L.S. (1998). Characterization of mannose receptor-dependent phagocytosis mediated by Mycobacterium tuberculosis lipoarabinomannan. Infect. Immun..

[47] Zamze S., Martinez-Pomares L., Jones H., Taylor P.R., Stillion R.J., Gordon S., Wong S.Y.C. (2002). Recognition of bacterial capsular polysaccharides and lipopolysaccharides by the macrophage mannose receptor. J. Biol. Chem..

[48] Schulert G.S., Allen L.A. (2006). Differential infection of mononuclear phagocytes by Francisella tularensis: Role of the macrophage mannose receptor. J. Leukoc. Biol..

[49] Ezekowitz R.A., Williams D.J., Koziel H., Armstrong M.Y., Warner A., Richards F.F., Rose R.M. (1991). Uptake of Pneumocystis carinii mediated by the macrophage mannose receptor. Nature.

[50] Lee S.J., Zheng N.Y., Clavijo M., Nussenzweig M.C. (2003). Normal host defense during systemic candidiasis in mannose receptor-deficient mice. Infect. Immun..

[51] Dan J.M., Kelly R.M., Lee C.K., Levitz S.M. (2008). Role of the mannose receptor in a murine model of Cryptococcus neoformans infection. Infect. Immun..

[52] Burgdorf S., Kautz A., Bohnert V., Knolle P.A., Kurts C. (2007). Distinct pathways of antigen uptake and intracellular routing in CD4 and CD8 T cell activation. Science.

[53] Martinez-Pomares L., Mahoney J.A., Kaposzta R., Linehan S.A., Stahl P.D., Gordon S. (1998). A functional soluble form of the murine mannose receptor is produced by macrophages *in vitro* and is present in mouse serum. J. Biol. Chem..

